# *Arnica Montana* L. Supercritical Extraction Optimization for Antibiotic and Anticancer Activity

**DOI:** 10.3389/fbioe.2022.897185

**Published:** 2022-05-10

**Authors:** Taja Žitek, Vesna Postružnik, Željko Knez, Andrej Golle, Barbara Dariš, Maša Knez Marevci

**Affiliations:** ^1^ Laboratory for Separation Processes and Product Design, Faculty of Chemistry and Chemical Engi-neering, University of Maribor, Maribor, Slovenia; ^2^ Faculty of Medicine, University of Maribor, Maribor, Slovenia; ^3^ National Laboratory for Health, Environment, and Food, Maribor, Slovenia

**Keywords:** mountain arnica, natural extract, active ingredients, antimicrobial activity, anticarcinogenic activity

## Abstract

*Arnica montana* L. flower heads are known for their antioxidant, antimicrobial, and anticancer activity. The aim of this work was to optimize the process of supercritical CO_2_ extraction, to achieve high extraction yield and high content of biologically active components, and to confirm the antimicrobial and anticancer activity of the extract. The influence of pressure and temperature on the total phenolic content, antioxidant activity, and proanthocyanidin content was evaluated. The pressure and temperature were found to be interdependent. A temperature of 60°C and a pressure of 30 MPa resulted in a high extraction yield, antioxidant activity and phenolic content. The content of proanthocyanidins was highest at a pressure between 18 and 24 MPa. The extracts inhibited three different microorganisms successfully; *Staphylococcus aureus, Escherichia coli* and *Candida albicans*, at concentrations ranging from 0.1 to 5.16 mg/ml and showed anticancer activity decrease up to 85% at a concentration of 0.5 mg/ml.

## 1 Introduction

*Arnica montana* is a protected plant species used widely in traditional and modern medicine for its antibacterial, antitumour, antioxidant, anti-inflammatory, and antifungal properties (Knuesel et al., 2002; Galambosi, 2004; Macêdo et al., 2004; Gawlik-Dziki et al., 2011; Craciunescu et al., 2012; Kowalski et al., 2015; Olioso et al., 2016; Kriplani et al., 2017; Sugier et al., 2019, 2020). The genus has more than 30 different species, and they differ significantly in the content of medicinal components, with *A. montana* being the species used most widely for its medicinal value (Kowalski et al., 2015; Kriplani et al., 2017) and the flower heads being the part of the plant containing the highest levels of biologically active compounds (Galambosi, 2004; Ganzera et al., 2008; Spitaler et al., 2008). The antimicrobial activity of the plant extracts has been studied extensively, as they have been shown to possess antimicrobial activity against a wide range of microorganisms (Brantner and Grein, 1994; Koo et al., 2000; Klaas et al., 2002; Iauk et al., 2003; Pljevljakušić, 2013; Bulfon et al., 2014; Kryvtsova, 2020). The anticancer activity of various components of *A. montana* has been investigated thoroughly, particularly sesquiterpene lactones, the main active compound of the plant (Lee et al., 1972; Lyß et al., 1998; Willuhn, 1998; Douglas et al., 2004; Huang P.-R. et al., 2005; Ghantous et al., 2010; Chaturvedi, 2011; Lim et al., 2012; Chadwick et al., 2013; Jakobs et al., 2016; Drogosz and Janecka, 2019; Kriplani and Guarve, 2020). The essential oils of the plant have been shown to possess cytotoxic activity on cells of anaplastic astrocytoma and glioblastoma multiforme (Sugier et al., 2019, 2020), while ethanol extracts of the plant also inhibited melanogenesis (Usui et al., 2015). Arnica plant contains more than 150 therapeutically active compounds (Ganzera et al., 2008; Kriplani et al., 2017), and in our search of the literature, we found no data published on the effect of plant extracts on the metabolic activity of cancer cells.

To prepare extracts from plant materials with safe, natural solvents, supercritical extraction (SCF) with carbon dioxide (CO_2_) was selected as the extraction method. In many studies, the method has been introduced as a pioneering technique for obtaining high-quality essential oils with minimal loss of its components, thus preserving its antimicrobial properties very well (Yousefi et al., 2019). Furthermore, the supercritical CO_2_ extract was found to contain high levels of active constituents, the sesquiterpenes, and to be stable in semisolid preparations (Bilia et al., 2006). The technique also represents an environmentally friendly process that can replace many ecologically harmful or potentially hazardous organic solvents (Raventós et al., 2002). It is becoming cost-effective on a production scale and is often used to extract natural materials that can be used in the food, pharmaceutical, and cosmetic industries (Soquetta et al., 2018).

The pharmaceutical and cosmetic industries need new formulations and active ingredients constantly. Extracts from natural materials represent a rich source of bioactive components that can be used for various applications. However, the literature mentions significant differences between natural extracts of the same material obtained by different extraction methods, conditions and solvents. Even within the same species, extracts vary considerably due to environmental and genetic factors. Therefore, investigations are encouraged of the differences between extracts from different material sources and extraction conditions (Talmaciu et al., 2015).

The aim of this study was to determine the optimal conditions (temperature and pressure) of SCF extraction to achieve the maximum extraction efficiency and content of active components (antioxidants, total phenols, and proanthocyanidins). The quality of the extract was evaluated by the determination of the content of biologically active components (antioxidant activity, phenolics and proanthocyanidins), and those with the highest proportion were subjected to further testing on their antimicrobial activity. The extract with the highest antioxidant activity was tested for cytotoxic activity against melanoma cells. This study describes the effect of temperature and pressure on the supercritical fluid extraction of Arnica. Optimum extraction conditions were determined with respect to the yield of biologically active compounds in the extract.

## 2 Materials and Methods

The *Arnica montana* was purchased from Alfred Galke (Samtgemeinde Bad Grund, Germany). The materials were supplied dried. The dried material was ground and then extracted. The extract was stored in the freezer until it was used for analysis. All analyses were performed within 1 month.

### 2.1 Design Expert Experimental Plan

The extraction process was designed and optimized using Design-expert Pro 12 (Stat-Ease, Inc., Minneapolis). Two independent factors, namely, a temperature between 35 and 64°C and pressure between 5.8 and 34.1 MPa, were selected for the experimental plan. Table 1 shows the experimental matrix for the supercritical extractions of *A. montana*.

**TABLE 1:** Two factor experimental design of supercritical extraction.

Sample	Temperature (°C)	Pressure (MPa)
1	36	20
2	64	20
3	40	30
4	50	34.1
5	50	20
6	40	10
7	60	30
8	60	10
9	50	5.9

### 2.2 Supercritical Fluid Extractions

The extractions were performed in an SFE system, as described (Žitek et al., 2020). The dried mixed flower head material (43 g) was placed in an autoclave with a volume of approximately 1,000 ml. The SFE was performed under various conditions determined by the design expert software, shown in the experimental plan (Table 1). The experimental parameters ranged in the pressure range from 6 to 34 MPa and temperatures from 35 to 65°C. The solvent flow rate and the amount of supercritical CO_2_ consumption were constant in all experiments. A high-pressure pump, a heater, an autoclave (produced by the company UHDE-GmbH-Hagen BRD), and a separator were used for the process. After the process, the extract was stored in a freezer until analysis.

### 2.3 Spectrophotometric Analyses

Ultraviolet spectrophotometric analyses were performed on the extracts to determine their antioxidant activity, total phenols, and proanthocyanidins. UV-VIS (BIOTEK SYNEGRY 2) apparatus was used for analysis.

#### 2.3.1 Determination of Antioxidant Activity

Antioxidant activity (AA) was determined by the 1,1-diphenyl-2-picrylhydrazyl (DPPH) method, as described (Huang D. et al., 2005). Briefly, 10 mg of the extract was weighed into a 10 ml volumetric flask, and methanol was added. The solution was mixed and dissolved completely in an ultrasonic bath. 3 ml of the DPPH solution and 77 μl of the extract solution were mixed and thermostatted for 15 min at room temperature. The absorbance was measured at 515 nm.

#### 2.3.2 Determination of Total Phenols

The total phenols were determined using an FC (Folin-Ciocateu) reagent, as described (Škerget et al., 2005). Briefly, 0.5 ml of the diluted extract was added to 2.5 ml FC reagent diluted 10-fold with distilled water, and 2 ml Na_2_CO_3_ (75 g/L) was added. The control was prepared using 0.5 ml of distilled water. A calibration curve was prepared using Galic Acid diluted in distilled water. The glass vials were thermostatted at 50°C for 5 min. The absorbance of cooled solutions was measured at 760 nm. The total phenolic content was expressed as mg GA per g of extract.

#### 2.3.3 Determination of Proantocianidins

The proanthocyanidins’ content was determined using iron(II) sulfate heptahydrate, hydrochloric acid, and butanol, as described (Škerget et al., 2005). Briefly, 77 mg of a solution [Fe (SO_4_) × 7H_2_O] was weighed into a 500 ml volumetric flask, and 500 ml of a 2:3 mixture of HCl and butanol was added. The extract was diluted with distilled water. 10 ml of iron(II) sulphate heptahydrate solution was added to the glass vial, mixed, and thermostatted in a water bath at T = 95°C for 15 min. The absorbance of the cooled samples was measured at a wavelength of 540 nm. The content of proanthocyanidins was expressed in mg PAC per g of extract.

### 2.4 Antimicrobial Activity of Extracts

The antimicrobial activity was determined for extracts with a high content of biologically active components. The extracts were tested for their *in vitro* antimicrobial activity against *Staphylococcus aureus* (MRSA) (ATCC 25923, ATCC, Wesel, Germany), *Escherichia coli* (ATCC 25922, ATCC, Wesel, Germany), and *Candida albicans* (ATCC 60193, ATCC, Wesel, Germany) using the broth microdilution method. Cation-adjusted MH broth supplemented with lysed horse blood and β-NAD (MH-F broth) was used as described (Žitek et al., 2020). A 96-well microtitre plate was filled with 100 μl of the MH broth and 100 μl of subsequently diluted extract. The concentration of the extract ranged from 18.750 mg/L to 0.037 mg/L. The positive control contained 50 μl of the MH broth and 50 μl of the diluted extract. The negative control contained 100 μl of MH broth and 10 μl of the microorganism (inoculum density of 10^8^ CFU/ml). After 24 h of incubation at 37°C, 30 μl of sterile 0.04% resazurin dye solution was added to each sample and incubated for an additional 4 h. Samples with bacterial growth discolored from dark blue to pink. The MIC endpoint was determined as the lowest drug concentration that resulted in a growth reduction of 90% or more compared to the negative control. All assays were performed in pentaplicate.

### 2.5 Anticancer Activity of Extracts on Melanoma Cells and Cell Apoptosis

Extracts with the highest content of biologically active components were tested for their *in vitro* cytotoxic activity against skin metastatic melanoma WM266-4 cells. The cells were grown in a complete medium with the following composition: 98.8% of Eagle’s Minimum Essential Medium (EMEM), 10% Foetal Bovine Serum (FBS), and 0.2% of MycoZap Plus-CL 500x. Cancer cells at a density of 1 × 10^4^ viable cells per well were incubated for 24 h in a 96 well culture plate to allow cell attachment. After that, the cells were exposed to *A. montana* extracts (c = 5; 4; 3; 2; 1; 0.5; 0.25; 0.1; 0.05; 0.01 mg/ml) for 24 h. The control cells were incubated in the medium without the added extract. The cells’ metabolic activity was measured spectrophotometrically with a colorimetric cell viability kit (WST 8, PromoKine, PromoCell, Heidelberg, Germany, EU). Absorbance was measured at 570 nm (630 nm background absorbance) in pentaplicates. The cells’ metabolic activity (MA) was calculated with the following equation:**%MA =**

$$\%MA=\frac{\left(A_{570}-A_{630}\right)\;test\;sample}{\left(A_{570}-A_{630}\right)\;control}\times 100$$ (1)

Where *A* is the average absorbance calculated from the pentaplicates. Cell morphology before and after the exposure of the cells to the extract was observed using an inverted microscope (DFC365 FX Leica, Buffalo Grove IL, United States).

A Muse Cell Analyzer and a Muse Annexin V and Dead Cell Kit (Luminex, Commercial Ave, Northbrook, IL) were used to examine apoptosis. Apoptosis was verified according to the manufacturer’s prescribed protocol Muse Annexin V and Dead Cell Kit catalog number MCH100105. After trypsinization of the cells, the cell suspensions were prepared for analysis. Briefly, Annexin V and dead cell reagent were added to each sample and mixed. The samples were analyzed using the Muse Cell Analyzer. Each experiment was performed in pentaplicate, and the mean value was determined.

## 3 Results and Discussion

### 3.1 Supercritical Fluid Extractions

Supercritical fluid extraction (SFE) was selected as the extraction method as it ensures efficient and rapid extraction, requires only moderate temperatures, eliminates the need for purification processes, and avoids the use of harmful organic solvents (Khaw et al., 2017). CO_2_ is used most widely as the solvent, as it is non-explosive, non-toxic, easily accessible, and easy to separate from the extracted products (Yousefi et al., 2019). The extraction conditions and equipment used in this study are similar to those recently used in industrustrial plants for isolation of active compounds. Based on the obtained extraction yields, a quadratic equation was fitted for calculating the efficiency of the supercritical extractions, with the lower limits of 10 MPa and 40°C and upper limits of 30 MPa and 60°C.

$${µ}_{\mathrm{extraction}}=-3.64795+0.236261\cdot T-0.001491\cdot p+0.000530\cdot T\cdot p-0.003544\cdot T^{2}-0.000038\cdot p^{2}$$ (2)

Where ${µ}_{extraction}$ is the extraction yield, T—the temperature at which the extraction takes place (°C), and p—the extraction pressure (bar). The equation shows that temperature and pressure are related. The model is significant (F = 70.29, *p* < 0.0001). The predicted squared Pearson coefficient (pred. R^2^) has a value of 0.8611, which is acceptable compared to the adjusted R^2^ (adj. R^2^) of 0.9665, as the difference between them is less than 0.2.

**FIGURE 1:**
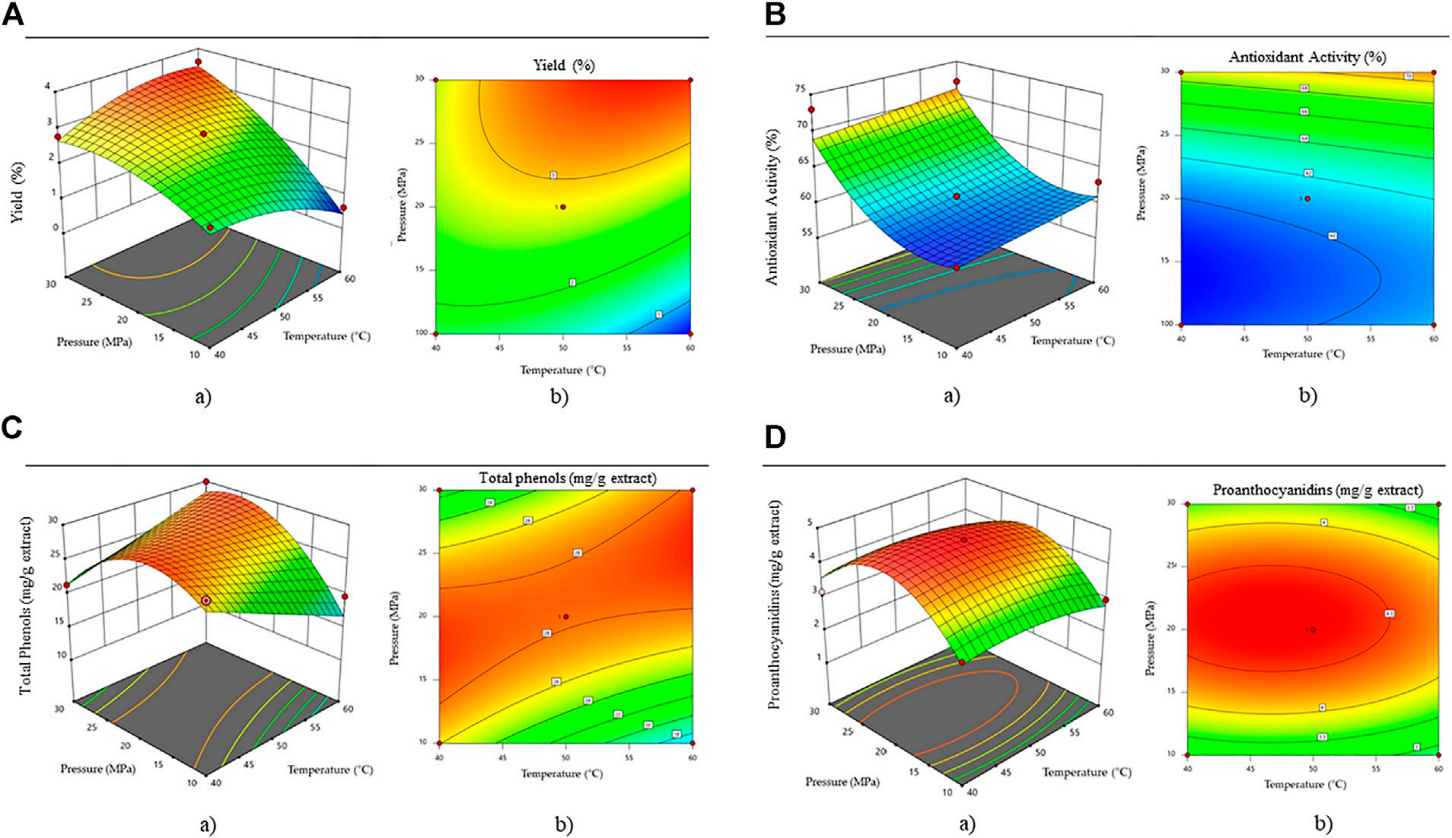
Effect of pressure and temperature on **(A)** efficiency, **(B)** antioxidant activity, **(C)** total phenolic content and **(D)** proanthocyanidine content. Where a) shows the three-dimensional response surface and b) Two-dimensional contour plot.

It can be seen from Figure 1A that pressure is a slightly more important parameter than temperature to achieve higher extraction efficiencies. The higher the pressure and temperature, the higher the extraction efficiency. The highest extraction efficiencies were obtained at a pressure above 30 MPa and a temperature above 50°C (yield = 3.5%).

The antioxidant activity of the obtained extracts is presented in Figure 1B. Using the design expert software a quadratic equation was fitted for the calculation of the antioxidants in the extracts, with the lower limits of 10 MPa and 40°C and upper limits of 30 MPa and 60°C.

$$\mathrm{AA}=61.54390+0.102695\cdot T-0.106778\cdot p+0.000392\cdot p^{2}$$ (3)

Where *AA* is the antioxidant activity in an individual extract (%), T—the temperature at which the extraction takes place (°C), and p—the extraction pressure (bar). The ANOVA analysis proved the model to be significant (F = 27.16, *p* < 0.0001). The pred. R^2^ has a value of 0.6726, which is acceptable compared to the adj. R^2^ of 0.8674. The highest antioxidant activity was obtained at the highest temperature (60°C) and pressure (30 MPa) conditions (AA = 73.72%). Ahmad et al. (2013) reported up to 71.52% DDPH scavenging activity for *A. montana* methanolic extracts.

Based on the total phenolic content measured in the extracts, a quadratic equation was established using the design expert software, with the lower limits of 10 MPa and 40°C and upper limits of 30 MPa and 60°C.

$$\mathrm{GA}=48.21307-0.876603\cdot T+0.016375\cdot p+0.004098\cdot T\cdot p-0.000511\cdot p^{2}$$ (4)

Where *GA* represents the total phenolic content in each extract, T—the temperature at which the extraction takes place (°C) and p—the extraction pressure (bar). The ANOVA analysis proved the model to be significant (F = 23.30, *p* = 0.0002). The pred. R^2^ has a value of 0.6816, which is acceptable compared to the adj. R^2^ of 0.8814. As seen in Figure 1C, the highest phenolic content was obtained at pressures between 14 and 22 MPa. From the point of view of the economy of the process, it is evident from the diagram that a temperature of 40°C and a pressure of 16 MPa would be sufficient for high values of total phenols. The content of total phenolics in extracts was decreased at pressures above 22 MPa due to the fact that under these conditions, the selectivity of the solvent probably decreases for the phenolic species. The polarity of the solvent mixture may become lower, and therefore, the solubility of polar phenolics is reduced.

The percentage of total phenolic acids in the extracts ranged from 1.31 to 3.25%. In comparison, Ganzera et al. (2008) reported from 1.03 to 2.24% of total phenolic acids, while Spitaler et al. (2008) reported from 1.32 to 2.35% of phenolic acids. In both studies, the extracts from the flower heads were prepared by ultrasonic extraction with methanol.

For proanthocyanidins’ content, a quadratic equation was established using the design expert software, with the lower limits of 10 MPa and 40°C and upper limits of 30 MPa and 60°C.

$$\mathrm{PAC}=-6.31204+0.238379\cdot T+0.052336\cdot p-0.002550\cdot T^{2}-0.000125\cdot p^{2}$$ (5)

Where *PAC* is the content of proanthocyanidins in each extract (mg/g extract). T—is the temperature at which the extraction takes place (°C) and p—is the extraction pressure (bar). The pred. R^2^ has a value of 0.8420, which is acceptable compared to the adj. R^2^ of 0.9502. The difference between them is less than 0.2. Figure 1D shows that too high a temperature or too high a pressure has a negative effect on the content of proanthocyanidins in the extracts. The highest values were obtained at pressures between 180 and 24 MPa, and temperatures between 37 and 55°C. To our knowledge, no studies have yet reported the content of proanthocyanidins in extracts of *A. montana*.

According to the surface response methodology and the ranges within the model equations (Eq. 5), optimal conditions for proanthocyanidins (Figures 1Da,b) in the extract were determined. In contrast to proanthocyanidins, the optimal extraction conditions (30 MPa and 60°C) resulted in the highest values for extraction yield, antioxidant activity, and phenolic components. Therefore, these dependent variables cannot yet be considered fully optimized, and further studies should investigate their content at operating conditions above the limits of this study. It is important to note that at conditions above this line, the characteristics of the solvent may change due to the varying process conditions and lower solubility of components of interest.

### 3.2 Antimicrobial Activity of the Extracts

The minimal inhibitory concentration (MIC) of the extracts of *A. montana* on gram-positive bacteria *S. Aureus*, gram-negative *E. coli*, and on yeast *C. albicans* are shown in Table 2. All the tested extracts inhibited microorganisms successfully, with *S. aureus* being the most sensitive and *C. albicans* the most resistant to the extract. The MIC values for *S. aureus* ranged from 0.10 to 0.31 mg/ml, for *E. coli* from 1.23 to 2.58 mg/ml, and for *C. albicans* from 1.41 to 5.16 mg/ml.

**TABLE 2:** Determination of MIC for *A. montana* extracts obtained by different extraction conditions against three different microorganisms.

Extraction conditions		MIC (mg/ml)		
**Temperature (°C)**	**Pressure (MPa)**	***S. aureus***	***E. coli***	***C. albicans***
40	30	0.10 ± 0.04	1.23 ± 0.60	4.69 ± 0.00
50	20	0.17 ± 0.09	2.34 ± 0.64	1.41 ± 0.57
60	30	0.31 ± 0.08	2.46 ± 0.57	4.69 ± 0.00
40	10	0.16 ± 0.08	2.58 ± 0.47	5.16 ± 0.94

Various effects of *A. montana* flower extracts on microorganisms have been reported in the literature. Antimicrobial activity against microorganisms used in this study has been previously confirmed for water (Brantner and Grein, 1994), ethanolic (Prelipcean et al., 2011; Kryvtsova, 2020) and methanolic extracts (Pljevljakušić, 2013; Kryvtsova, 2020; Nieto-Trujillo et al., 2021), but this study was the first to confirm antimicrobial activity for SCF-CO_2_ extract. Brantner and Grein determined a MIC of 16.7 mg/ml for aqueous flower extracts against *S. aureus* and *E. coli* (Brantner and Grein, 1994), while Pljevljakušić, (2013) reports a MIC of 5 μl/ml for methanolic extracts for both bacteria, while *C. albicans* showed greater resistance with MIC values of up to 83 μl/ml. These results are consistent with studies reporting the effect of extracts on microbial growth. Both Prelipcean et al. (2011) and Nieto-Trujillo et al. (2021) found greater inhibition of *A. montana* extract against *S. aureus* compared to *E. coli*, while Prelipcean et al. (2011) confirmed higher resistance of *C. albicans*.

Plant alcoholic extracts inhibit a range of other genera not included in this study, from gram-positive *Actinomyces* spp.*, Bacillus* spp.*, Listeria* sp.*, Micrococcus* spp. *and Peptostreptococcus* sp. to gram-negative *Acinetobacter* sp.*, Capnocytophaga* sp.*, Eikenella* sp.*, Fusobacterium* sp.*, Klebsiella* sp.*, Listonella* sp.*, Photobacterium* sp.*, Porphyromonas* sp.*, Prevotella* sp.*, Pseudomonas* sp.*, Salmonella* sp. and *Veillonella* sp. (Koo et al., 2000; Iauk et al., 2003; Prelipcean et al., 2011; Pljevljakuši, 2013; Bulfon et al., 2014; Kryvtsova, 2020).

A thorough search of the relevant literature yielded two related studies that reported no antimicrobial activity against *S. aureus* and *E. coli* for ethanolic extracts (Koo et al., 2000) and essential oils prepared with steam distillation (Miller et al., 2015). However, extracts showed inhibition against *Actinomyces naeslundii, Porphyromonas gingivalis and Streptococcus mutans* (Koo et al., 2000; Miller et al., 2015).

The MIC of the extracts described in this study, prepared by supercritical extraction with CO_2_, are similar to those previously described by conventional extractions. The MIC of antibiotics is 0.25–4 μg/ml for *S. aureus* and 0.008–2 μg/ml for *E. coli*. Rubin et al. (2011) reported MIC against *S. aureus* at 0.25 μg/ml for penicillin and at 4 μg/ml for ciprofloxacin. Pohl et al. (2018) reported MIC against *E. coli* between 0.008 and 0.03 μg/ml for penicillin and between 0.5 and 2 μg/ml for ciprofloxacin. According to Eksi et al. (2013), the MIC against *C. albicans* is between 0.03 and 32 μg/ml for four different fungicides.

### 3.3 Anticancer Activity of the Extracts on Melanoma Cells and Cell Apoptosis

**FIGURE 2:**
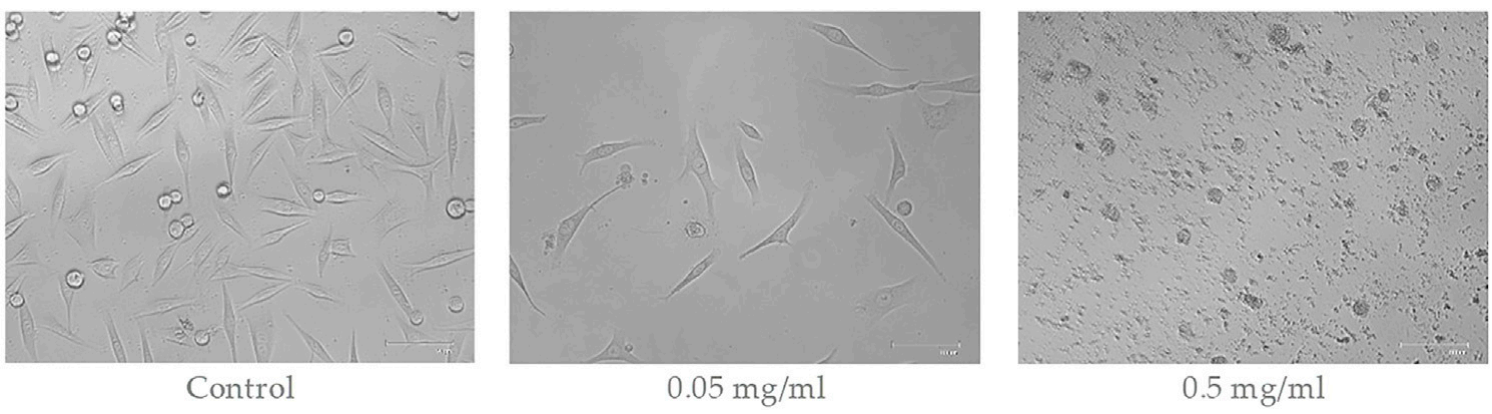
Morphological changes of melanoma WM-266-4 cells after exposure to different concentrations of *A. montana* extract.

Extracts of *A. montana* have shown cytotoxic activity against melanoma cells WM-266-4. Figure 2 shows the cell morphology 24 h after exposure to the *A. montana* extract. The untreated control group showed typical cell morphology with a dendritic phenotype. A decrease in cell density was observed with the increasing concentration of the extract. At an extract concentration of 0.5 mg/ml and above, cytotoxicity was clearly observed as cells condensed and disintegrated into apoptotic bodies.

**FIGURE 3:**
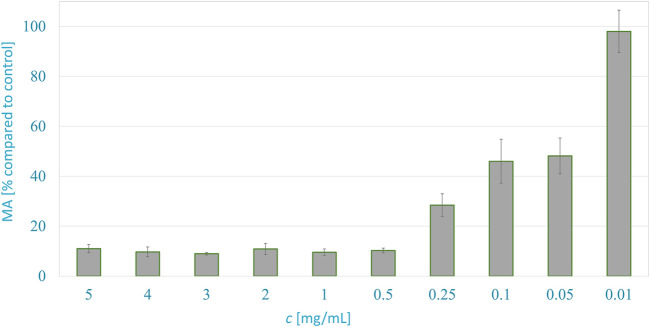
Anticancer activity of *A. montana* extract against melanoma WM266-4 cells, 24 h after exposure to various concentrations.

Figure 3 shows the metabolic activity of WM-266-4 melanoma cells in dependence on the *A. montana* extract concentration 24 h after exposure. The metabolic activity of cancer cells was inhibited successfully by extracts at concentrations ranging from 5 to 0.5 mg/ml. The metabolic activity of cancer cells decreased to about 14% compared to control with typical cell morphology. The half-maximal effective concentration (EC_50_) of the extract was just below the concentration of 0.05 mg/ml, as the cells had 48.2% MA compared to the control.

The significance, as well as the variability of the results, were confirmed by an experiment on normal human epidermal melanocytes. The results show that the metabolic activity decreased by 7% after the application of the extract, which is still within the range of deviations when the control results are repeated. According to the results, the application of the extract has no significant effect on healthy cells.

**FIGURE 4:**
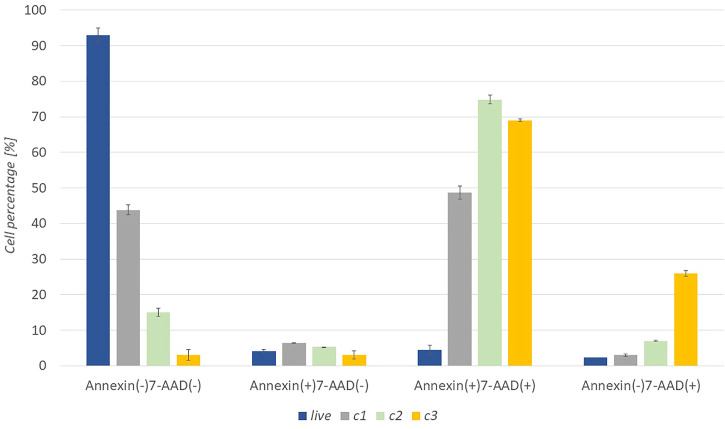
Apoptotic activity of A. montana extract against melanoma WM-266-4 cells at various extract concentrations (c_1_ = 0.1 mg/mL, c_2_ = 0.25 mg/mL, c_3_ = 0.5 mg/mL).

Cell death apoptosis was also studied to support the metabolic activity of cancer cells. The response of cells to the extract was studied in more detail at three concentrations (*c*_1_ = 0.1 mg/ml, *c*_2_ = 0.25 mg/ml, *c*_3_ = 0.5 mg/ml). After 24 h of incubation of the cells with the extract, the cells were stained with Muse™ Annexin V & Dead Cell Reagent and recorded with Muse™ Cell Analyzer. The graph in Figure 4 shows a representative test result with untreated WM-266-4 cells and WM -266-4 cells treated with extracts of concentrations c_1_, c_2,_ and c_3_. Where the percentage of live [Annexin (−) 7-AAD (−)], early apoptotic [Annexin (+) 7-AAD (−)], late apoptotic [Annexin (+) 7-AAD (+)] and cellular residues [Annexin (−) 7-AAD (+)] can be seen.

Apoptotic results show that untreated cells reach high levels in the Annexin (−) 7-AAD (−) range, which represents healthy, living cells. Whereas after the application of c_1_ extract, about 50% of the cells fell in the Annexin (+) 7-AAD (+) range, which represents late apoptosis. Thus, we can conclude that the concentration of c_1_ already inhibited the function of half of the cells. However, the application of the extract with a higher concentration (c_2_ and c_3_) strongly suppressed the cells. At c_2,_ the highest percentage of cells were in the area of Annexin (+) 7-AAD (+). Similar results are evident for c_3,_ however debris [Annexin (−) 7-AAD (+)] was significantly higher than at other concentrations.

## 4 Conclusion

In this study, the optimum conditions for supercritical extraction of *A. montana* were determined to obtain the highest extraction yield and biologically active component content. Quadratic models were fitted successfully for *A. montana* extraction yield, antioxidant activity (AA) and total phenol (GA) content. While high temperature and pressure conditions resulted in high extraction yields and antioxidant activity, the total phenol content decreased at pressure conditions above 22 MPa. Similarly, for the proanthocyanidins, moderate pressure and temperature conditions were also better.

The prepared extracts with the highest content of biologically active ingredients were effective in inhibiting all the tested microorganisms. Significant inhibition of gram-positive *S. aureus* was detected at an MIC of 0.10 mg/ml–0.31 mg/ml. The supercritical extract obtained at 40°C and 30 MPa inhibited bacteria at a MIC of 0.10 mg/ml. For gram-negative *E. coli*, the MIC values ranged from 1.23–2.58 mg/ml. However, the lowest MIC was required by the same extract as for *S. aureus* (40°C, 30 MPa). The fungus *C. albicans* (MIC = 1.41 mg/ml–5.16 mg/ml) was also inhibited by all the extracts. The extract prepared at 50°C and 20 MPa required the lowest concentration (1.41 mg/ml). The MIC of the prepared extracts is comparable to the MIC of antibiotics used against *S. aureus* and *E. coli,* and of fungicides used against *C. albicans*. This is confirmation of the potential of the extract for use as an equivalent to topical antibiotics.

This study also confirmed that the prepared extracts exerted an anticancer effect through inhibition of melanoma cell metabolic activity. This indicates a need for further research of extract’s molecular structure and component implementation, individually and in combination, for the development of antibiotic and melanoma therapy.

## Data Availability

The original contributions presented in the study are included in the article/Supplementary Material, further inquiries can be directed to the corresponding author.
