# Supplementary figures and images for: Arnica Montana L. Supercritical Extraction Optimization for Antibiotic and Anticancer Activity
Source: Front Bioeng Biotechnol. 2022 May 10;10:897185. doi: 10.3389/fbioe.2022.897185 (PMC9127360; doi:10.3389/fbioe.2022.897185)

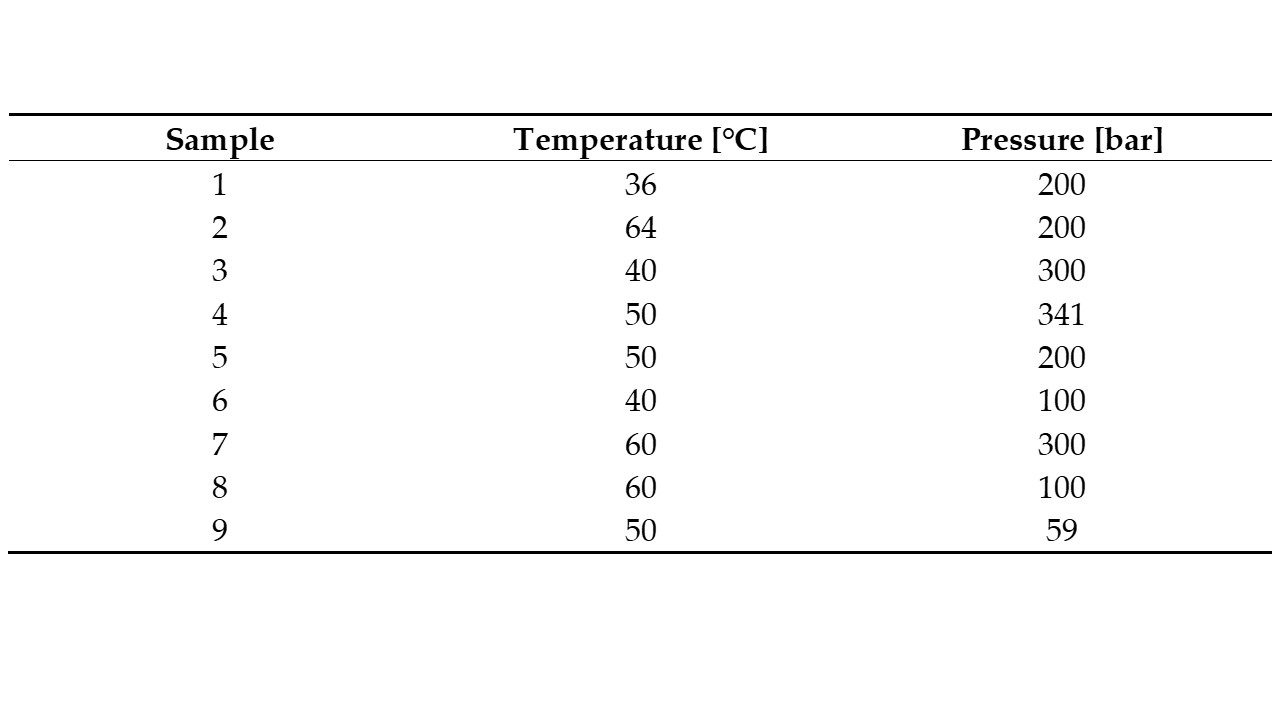

Supplement: Supplementary file 1 [file Image1.JPEG]

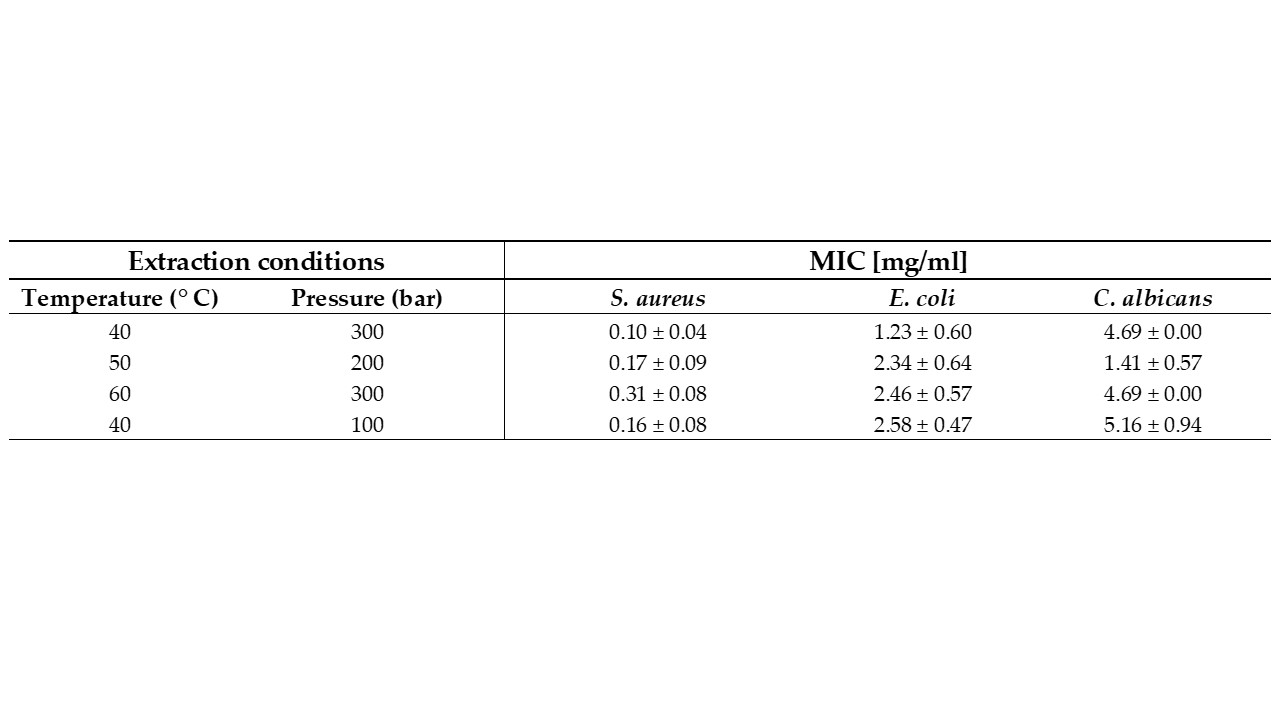

Supplement: Supplementary file 2 [file Image2.JPEG]
